# Substituted titanocenes induce caspase-dependent apoptosis in human epidermoid carcinoma cells *in vitro* and exhibit antitumour activity *in vivo*

**DOI:** 10.1038/sj.bjc.6604021

**Published:** 2007-10-09

**Authors:** J H Bannon, I Fichtner, A O'Neill, C Pampillón, N J Sweeney, K Strohfeldt, R W Watson, M Tacke, M M Mc Gee

**Affiliations:** 1 UCD School of Biomolecular and Biomedical Science, Conway Institute of Biomolecular and Biomedical Research, University College Dublin, Belfield Dublin 4, Ireland; 2 Max Delbrück Center (MDC) for Molecular Medicine, Robert-Rössle-Straße 10, Berlin D-13092, Germany; 3 UCD School of Medicine and Medical Science, Conway Institute of Biomolecular and Biomedical Research, University College Dublin, Belfield Dublin 4, Ireland; 4 UCD School of Chemistry and Chemical Biology, Centre for Synthesis and Chemical Biology (CSCB), Conway Institute of Biomolecular and Biomedical Research, University College Dublin, Belfield Dublin 4, Ireland

**Keywords:** titanocene, epidermoid cancer, apoptosis, caspases, chemotherapy, cisplatin

## Abstract

Titanocene compounds are a novel series of agents that exhibit cytotoxic effects in a variety of human cancer cells *in vitro* and *in vivo*. In this study, the antiproliferative activity of two titanocenes (Titanocenes X and Y) was evaluated in human epidermoid cancer cells *in vitro*. Titanocenes X and Y induce apoptotic cell death in epidermoid cancer cells, with IC50 values that are comparable to cisplatin. Characterisation of the cell death pathway induced by titanocene compounds in A431 cells revealed that apoptosis is preceded by cell cycle arrest and the inhibition of cell proliferation. The induction of apoptosis is dependent on the activation of caspase-3 and -7 but not caspase-8. Furthermore, the antitumour activity of Titanocene Y was tested in an A431 xenograft model of epidermoid cancer. Results indicate that Titanocene Y significantly reduced the growth of A431 xenografts with an antitumour effect similar to cisplatin. These results suggest that titanocenes represent a novel series of promising antitumour agents.

Cisplatin is one of the most effective chemotherapeutic agents known, displaying clinical activity against a wide range of human tumours, including cancer of the ovary, testis, bladder, lung, head and neck, cervix and endometrium and as a second-line therapy in the treatment of breast cancer (Farrell *et al*, 2004). The cytotoxic effect of cisplatin is thought to be mediated by its interaction with DNA to form DNA adducts and intrastrand crosslink adducts, which activate several signal transduction pathways that culminate in the induction of apoptotic cell death (Siddik, 2003).

Despite the success of cisplatin in the treatment of cancer, it is frequently associated with severe toxicity, such as severe nausea, vomiting, diarrhoea, renal and nerve toxicity. However, despite this, the antineoplastic effects of cisplatin has generated much interest in developing new organometallic complexes with reduced toxicity and improved clinical activity (Reedijk, 2003). This led to the development of alternative transition metal-based anticancer drugs (Farrell *et al*, 1999; Gelasco and Lippard, 1999; Jamieson and Lippard, 1999); however, success in this area has been slow. One such group, the metallocene dichlorides show remarkable antitumour activity (Köpf-Maier and Köpf, 1987, 1998); however, unfortunately, the efficacy of titanium dichloride (Cp_2_TiCl_2_) in phase II clinical trials in patients with metastatic renal (Lummen *et al*, 1998) or breast cancer (Kröger *et al*, 2000) was too low to pursue. This highlights the requirement for additional agents in the treatment and management of human cancer. In recent years, combination regimens involving cisplatin and microtubule-disrupting agents have been explored, and initial results demonstrate improved response rates over single therapy regimens (Rose *et al*, 1999; Sawada *et al*, 2003).

Apoptosis can occur through a number of signalling pathways broadly classified as the extrinsic and intrinsic cascades. A common component of these two pathways is a family of cysteine proteases known as caspases that become activated following cleavage by upstream activators. Caspases activate downstream substrates ultimately leading to the regulated dismantling of the cell. This tightly regulated process differs greatly from necrotic cell death, which occurs as a result of non-specific cell injury. In this case, the cells swell and eventually rupture releasing their contents into the surrounding tissue, and thereby eliciting an inflammatory response (Vermeulen *et al*, 2005).

*Ansa*-Titanocenes are a novel series of drugs synthesised from titanium dichloride and fulvenes (Teuber *et al*, 1997; Hartl *et al*, 2001; Tacke *et al*, 2001b; Siddik, 2003) that contain highly substituted ethylene bridge (Tacke *et al*, 2001a, 2004; Rehmann *et al*, 2005). The subsequent synthesis of unbridged titanocene analogues led to increased cytotoxicity, suggesting a promising structure–function activity of these compounds (Sweeney *et al*, 2005, 2006). Two members of this series, [1,2-di(cyclopentadienyl)-1,2-di-(4-*N*,*N*-dimethylaminophenyl)ethanediyl] titanium dichloride and bis-[(*p*-methoxybenzyl)cyclopentadienyl] titanium(IV) dichloride, known as Titanocenes X and Y, respectively (Figure 1), display antiproliferative and cytotoxic effects in a variety of tumour cells. The antiproliferative activity of Titanocenes X and Y was studied in 36 human tumour cell lines and compared to cytotoxicity induced by the conventional agent cisplatin (Kelter *et al*, 2005). It was found that the highly substituted titanocenes are as effective as cisplatin at inducing cell death against a broad range of human tumours, and in some cases, titanocenes displayed greater cytotoxicity than cisplatin. Titanocenes significantly reduce growth of *ex vivo* tumour explants (Oberschmidt *et al*, 2005) and reduce tumour growth *in vivo* (Fichtner *et al*, 2006; Valadares *et al*, 2006; Beckhove *et al*, 2007). Collectively, these reports suggest the potential of highly substituted titanocenes as novel anticancer agents in single or combination treatment regimens and for treatment of cisplatin-resistant tumours. However, to date, the mechanism of action of titanocenes remains unknown, and further studies are required to improve our understanding of the precise mechanism of action of these novel compounds. This is essential to progress the search and development of new antitumour drugs.

We have investigated cell cycle and cell death alterations induced by titanocenes in HeLa cervical epithelial carcinoma cells and in the A431 human epidermoid carcinoma cell line. We have found that cell cycle alterations lead to the activation of caspases and cell death through apoptosis. This provides valuable insights into the mechanism of action of novel titanocene agents. Furthermore, we report encouraging *in vivo* results that further highlight the potential of substituted titanocenes for the treatment of cancer.

## MATERIALS AND METHODS

### Materials

[1,2-Di(cyclopentadienyl)-1,2-di-(4-*N*,*N*-dimethylaminophenyl) ethanediyl] titanium dichloride (Titanocene X), bis-[(*p*-methoxybenzyl) cyclopentadienyl] titanium(IV) dichloride (Titanocene Y) and Cp_2_TiCl_2_ were obtained from UCD School of Chemistry and Chemical Biology (Dublin, Ireland). The caspase-3, -7 and -8 antibodies were from Cell Signalling Technologies (Danvers, MA, USA). Glyceraldehyde 3-phosphate dehydrogenase (GAPDH) and *β*-actin antibodies were from Sigma-Aldrich (Dublin, Ireland). Unless otherwise stated, all other chemicals were purchased from Sigma-Aldrich. Annexin V/FITC antibody was from BD Biosciences, Oxford, UK. The HeLa cervical carcinoma cell line and the A431 carcinoma cell line were purchased from the American Tissue Culture Collection, LGC Promochem, Middlesex, UK.

### Methods

#### Growth inhibition assay

HeLa and A431 cells (1 × 10^4^) were seeded in 0.1 ml growth medium (MEM supplemented with 10% FCS, 2 mM L-glutamine and 100 *μ*g ml^−1^ penicillin/streptomycin) in a 96-well plate and incubated overnight at 37°C. Cells were treated with vehicle (0.5% (v/v) DMSO), or either Titanocene X, Titanocene Y, Cp_2_TiCl_2_ and cisplatin in a concentration range of 2.5 × 10^−4^–5 × 10^−8^ M and incubated at 37°C for 24 and 48 h. A volume of 20 *μ*l of MTT solution (3-(4,5-dimethylthiazol-2-yl)-2-5-diphenyltetrazolium bromide) (5 mg ml^−1^ in PBS) was added, and plates were incubated at 37°C in the dark for 4 h. Growth medium was removed and replaced with DMSO (200 *μ*l) to dissolve the blue formazan crystals formed. Results were recorded spectrophotometrically at 540 nm.

#### Cell cycle profile

HeLa and A431 cells (1 × 10^6^) were treated with the indicated compounds and incubated at 37°C. Following treatment the cells were harvested by trypsination, washed in PBS and fixed overnight in ethanol (70% (v/v)) at 4°C. Following incubation, the ethanol was removed and the cells were resuspended in PBS (400 *μ*l). RNase A (10 *μ*g ml^−1^) was added and DNA was stained with propidium iodide (0.2 mg ml^−1^ in PBS) and incubated in the dark at 37°C for 30 min. The DNA content of 10 000 cells was analysed by Flow cytometry.

#### Annexin V and propidium iodide staining

A431 cells (0.8 × 10^6^) were treated with the indicated compounds and incubated at 37°C. Following treatment, the cells were harvested by trypsination and centrifugation at 1000 r.p.m. for 5 min. The supernatant was removed, and the cell pellet was washed in PBS followed by two washes in binding buffer (10 mM HEPES, 150 mM NaCl, 5 mM KCl, 1.8 mM CaCl_2_, 1 mM MgCl_2_). The cells were incubated with an Annexin V/FITC antibody (5 *μ*l in 100 *μ*l binding buffer) and incubated at 4°C for 15 min in the dark. Samples were washed in binding buffer, and the supernatant was discarded. The pellet was resuspended in 490 *μ*l binding buffer and 10 *μ*l propidium iodide (10 mg ml^−1^ in PBS) was added to the samples before analysis by flow cytometry.

#### A431 xenografts

The *in vivo* experiments consist of three groups of mice treated with co-solvent, Titanocene Y and cisplatin, respectively. A431 cells (1 × 10^7^) were injected subcutaneously (s.c.) into female NMRI:nu/nu mice (eight mice per group). Tumours reached a palpable size of 5–6 mm diameter before treatment was initiated. Titanocene Y was dissolved in DMSO (final concentration 10%) and diluted with 0.5% Tween 80 in saline. The mixture was injected intraperitoneally (i.p.) at a dose of 40 mg kg^−1^ day^−1^ (predetermined) once daily for 5 consecutive days. One group of mice was treated with solvent (negative control), whereas the remaining group was treated with cisplatin (Medac GmbH, Hamburg, Germany) at a dose of 3 mg kg^−1^ day^−1^ for 5 consecutive days. Tumour size was measured using callipers, and tumour volume and relative tumour volume (in relation to the first treatment day) in the three experimental groups was calculated. Body weight and morbidity of the mice were determined continuously during the experiments for an estimation of tolerability. The animal experiment was performed according to the German Animal Protection Law and with approval from the responsible authorities. The *in vivo* procedures were consistent and in compliance with the UKCCCR guidelines.

#### Western blotting

A431 cells (1 × 10^6^) were treated with the indicated compounds as outlined. The cells were harvested by trypsination and centrifugation at 1000 r.p.m. for 5 min. The resulting pellet was washed in PBS and lysed in 100 *μ*l urea lysis buffer (7 M urea, 2 M thiourea, 2% CHAPS, 1% DTT, 0.8% pharmalyte, one tablet of complete protease inhibitors (per 20 ml lysis buffer) and incubated on ice for 10 min followed by centrifugation for 5 min at 13 000 r.p.m. to pellet cell debris. Protein content was determined using a Bradford assay. Protein was resolved by SDS–PAGE, transferred onto PVDF membrane and blocked overnight at 4°C in TBS containing 5% (w/v) dried milk followed by incubation with primary antibody for 1 h at room temperature. The membrane was washed with TBS containing 0.1% (w/v) Tween 20 and incubated with horseradish peroxidase-conjugated secondary antibody for 1 h and enhanced chemiluminescence was used to visualise the proteins.

## RESULTS

### Dose- and time-dependent cytotoxic effect of titanocene compounds in HeLa and A431 cells

**Figure 2:**
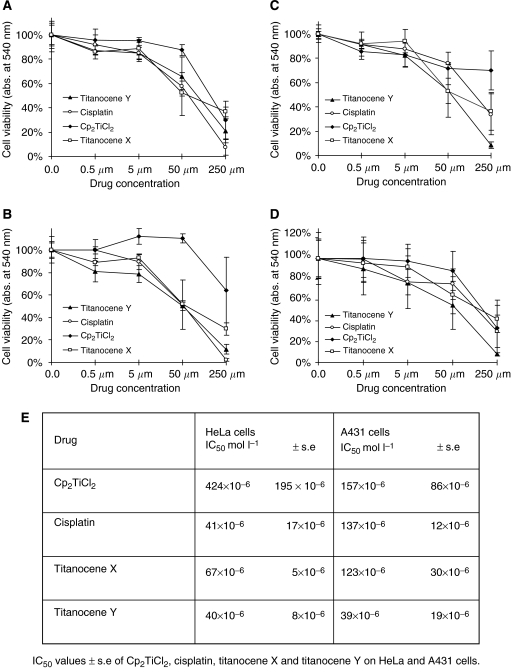
Antiproliferative effect of Titanocenes X and Y, Cp_2_TiCl_2_ and cisplatin on HeLa and A431 cells. HeLa (**A** and **B**) and A431 (**C** and **D**) cells were treated with vehicle (DMSO (0.5% v/v)) or 0.5, 5, 50 and 250 *μ*M of the indicated compound for 24 h (**A** and **C**) and 48 h (**B** and **D**). Cell viability was determined using an MTT assay and absorbance was monitored spectrophotometrically at 540 nm. Results represent the mean±s.e.m. of three separate experiments. (**E**) IC50 values±s.e. from Cp_2_TiCl_2_, cisplatin, Titanocenes X and Y treatment of HeLa and A431 cells for 48 h, calculated using the PRISM statistical analysis software package.

The *in vitro* cytotoxicity of Titanocenes X and Y, Cp_2_TiCl_2_ and cisplatin was determined in HeLa and A431 cells. Cells were treated with 0.5, 5.0, 50 or 250 *μ*M of each drug for up to 48 h. Results shown in Figure 2 outline that all compounds induced a varying degree of cytotoxicity in HeLa and A431 cells. Importantly, it was found that Titanocene X-induced cytotoxicity was greater than that induced by Cp_2_TiCl_2_ and similar to that induced by cisplatin in the two cell lines. Titanocene Y-induced cytotoxicity was significantly greater than that observed following Cp_2_TiCl_2_ treatment and similar to cisplatin in cells, however, Titanocene Y induced greater cytotoxic effect than both Cp_2_TiCl_2_ and cisplatin in A431 cells. The IC50 values have been calculated for each compound based on 48-h survival assays (Figure 2E). Cp_2_TiCl_2_ has an IC50 value of 424 × 10^−6^ mol l^−1^ when tested on the HeLa cells, whereas cisplatin, Titanocenes X and Y have significantly lower IC50 values of 41 × 10^−6^, 67 × 10^−6^ and 40 × 10^−6^ mol l^−1^, respectively. Cp_2_TiCl_2_ has an IC50 value of 157 × 10^−6^ mol l^−1^ in A431 cells, whereas cisplatin and Titanocene X have similar IC50 values of 137 × 10^−6^ and 123 × 10^−6^ mol l^−1^, respectively. However, Titanocene Y induced significantly greater loss in cell viability in this cell line over Titanocene X, cisplatin and Cp_2_TiCl_2_, with an IC50 value of 39 × 10^−6^ mol l^−1^. Loss of cell viability was observed with 0.5 *μ*M Titanocene Y. This data may highlight an important cytotoxic advantage of Titanocene Y over the traditionally used cisplatin in cancer treatment.

### Titanocene Y efficiently reduces A431 xenograft growth *in vivo*

**Figure 3:**
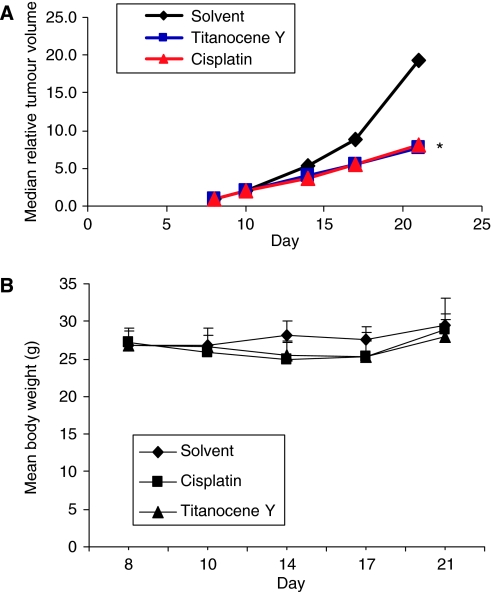
Effect of Titanocene Y and cisplatin on growth of A431 xenograft mice *in vivo.* A431 cells (1 × 10^7^) were injected subcutaneously (s.c.) at day 0 into female NMRI:nu/nu mice (eight per group) allowed to reach 5–6 mm in diameter. Mice were treated i.p. with cisplatin (3 mg kg day^−1^) or Titanocene Y (40 mg kg day^−1^) for 5 consecutive days starting on day 8. For each treatment group, tumour volume was measured using calipers on days 10, 14, 17 and 21 (**A**) and mean body weight was calculated (**B**).

In this study, we found that Titanocene Y is more potent than Titanocene X at inducing cell death of HeLa and A431 cancer cells *in vitro*. We have expanded the study to an *in vivo* xenograft A431 mouse model using the more potent compound, Titanocene Y, and we compared the effects observed to cisplatin when treated at their respective maximum tolerable dose, which we have previously determined as 40 mg kg day^−1^ and 3 mg kg^−1^ day^−1^, respectively (Fichtner *et al*, 2006). Three groups of mice consisting of eight mice each were treated i.p. with solvent, Titanocene Y (40 mg kg^−1^ day^−1^) or cisplatin (3 mg kg day^−1^) for 5 consecutive days, and tumour size was measured twice weekly for 3 weeks. As shown in Figure 3A, Titanocene Y induced a significant inhibition of tumour growth, with 40% inhibition in mean tumour volume following drug treatment in comparison to control animals. From this study, it is apparent that Titanocene Y is as good as cisplatin at delaying growth of A431 xenografts *in vivo*. The tumour growth in the mice treated with cisplatin displayed a similar trend with a 41% reduction of mean tumour volume detected following drug treatment in comparison to control animals. In the two mice cohorts treated with Titanocene Y and cisplatin, a moderate body weight loss was observed, representing 8 and 5%, respectively (Figure 3B).

### Titanocene Y induces apoptotic cell death in A431 cells

**Figure 4:**
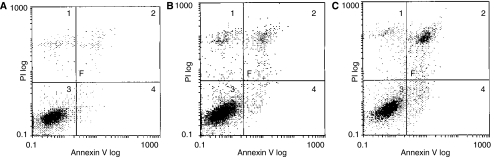
A431 cells undergo apoptosis when treated with Titanocene Y. Cells (0.8 × 10^6^) were treated with (**A**) vehicle (DMSO (0.5% v/v)) or (**B**) Titanocene Y (50 *μ*M) or (**C**) cisplatin (50 *μ*M) for 48 h. Cells were harvested by trypsination and centrifugation and stained with Annexin V/FITC antibody for 15 min according to the manufacturer's instructions. Cells were washed in binding buffer and stained with propidium iodide. Annexin V and propidium iodide fluorescence was measured by flow cytometry.

Cisplatin has been shown to induce apoptotic cell death in human cancer cells, including A431 cells (Gonzalez *et al*, 2001; Cummings and Schnellmann, 2002; Sawada *et al*, 2003). To investigate the mechanism by which Titanocene Y induces cell death, A431 cancer cells were stained with Annexin V and propidium iodide to detect early and late apoptotic cells. Vehicle-treated cells contained negligible levels (3%) of apoptotic cells, whereas treatment with Titanocene Y and cisplatin induced 18 and 23% apoptosis, respectively (Figure 4), confirming that cell death occurs via apoptosis.

### Titanocenes X and Y induce cell cycle arrest in A431 cells

**Figure 5:**
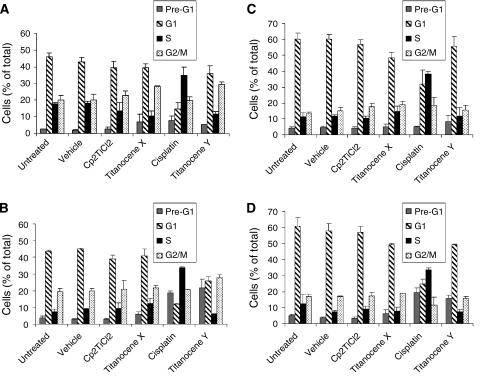
Effect of Cp_2_TiCl_2_, cisplatin, Titanocene X and Titanocene Y on A431 and HeLa cell cycle. A431 (**A** and **B**) and HeLa (**C** and **D**) cells were treated with vehicle (DMSO) (0.5% v/v) or 50 *μ*M Cp_2_TiCl_2_, cisplatin, Titanocene X or Titanocene Y for (**A** and **C**) 24 h and (**B** and **C**) 48 h. DNA was stained with propidium iodide, and DNA content was analysed by flow cytometry. Results represent the mean±s.e.m. of three separate experiments.

The mechanism of action of titanocenes in tumour cells is unknown. To investigate their mechanism of action, the effect of titanocenes on cell cycle progression of A431 and HeLa cells was analysed, and this was compared to Cp_2_TiCl_2_ and cisplatin. As shown, untreated and vehicle-treated cells displayed a normal cell cycle profile at 24-h (Figures 5A and C) and 48-h (Figures 5B and D) following treatment. In contrast, treatment of A431 and HeLa cells with cisplatin results in cell cycle arrest at S phase. Cell cycle arrest, which is apparent following a 24-h treatment with the drug, precedes the appearance of a pre-G_1_ peak at 48 h that is indicative of apoptosis in these cells. As indicated earlier, Cp_2_TiCl_2_ has little effect on A431 and HeLa cells and did not alter the cell cycle profile significantly at 24 or 48 h after treatment. An increase in cells in G_2_/M was observed following Titanocene X treatment in the A431 cells, but was less apparent in the HeLa cells. In contrast, Titanocene Y caused the accumulation of cells in G_2_/M phase with a concomitant decrease of cells in G_1_ phase of the A431 cell cycle at 24 h (Figure 5A), which precedes the appearance of a pre-G_1_ peak at 48 h (Figure 5B). While cell cycle arrest was not apparent in HeLa cells following treatment with Titanocene Y, an increased pre-G_1_ peak was apparent at 48 h following treatment (Figure 5D). Overall, the cell cycle alterations observed with Titanocenes X and Y differ from that observed following cisplatin treatment in A431 cells, and suggests that cisplatin and Titanocene compounds initiate their apoptotic effects via alternative mechanisms.

### Titanocene Y induces caspase activation in A431 cells

**Figure 6:**
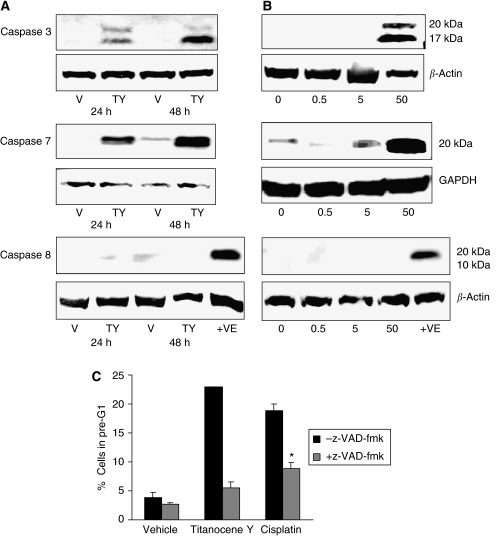
Western blot analysis of caspase-3, -7 and -8 following Titanocene Y treatment of A431 cells. Cells were treated with either vehicle (0.5% DMSO (v/v)) or Titanocene Y (50 *μ*M) for (**A**) 24 or 48 h or (**B**) a range of Titanocene Y concentrations (0–50 *μ*M) for 48 h. Whole cell extracts were prepared and protein (60 *μ*g) and resolved by SDS–PAGE followed by Western blotting using antibodies to detect the active cleavage product of caspase-3, -7 and -8. As a positive control (+VE) for caspase 8 activity, PWR-1 prostate cells were treated with etoposide (10 *μ*M) for 48 h. Blots were stripped and re-probed with *β*-actin or GAPDH as loading controls. Results are representative of three independent experiments. (**C**) A431 cells (1 × 10^5^ cells) were treated with vehicle (DMSO (0.5% v/v)), 50 *μ*M Titanocene Y or 50 *μ*M cisplatin for 48 h, or a pretreatment of z-VAD-fmk (150 *μ*M) for 1 h prior to DMSO (0.5% v/v) or 50 *μ*M Titanocene Y or cisplatin for a further 48 h. Cell viability was determined by flow cytometry following propidium iodide staining. The appearance of a pre-G_1_ peak represents non-viable cells. ^*^P<0.005 with respect to cisplatin treatment, Student's *t*-test. Results represent the mean±s.e.m. of three separate experiments.

It has been previously reported that cisplatin-induced apoptosis in A431 cells is mediated by the activation of caspases (Shapiro and Harper, 1999). To characterise the apoptotic cell death pathway activated by the most potent titanocene compound (Titanocene Y) in A431 cells, we investigated the activity of caspases. Cells were treated with Titanocene Y (50 *μ*M) for 24 and 48 h or a range of titanocene concentrations (0.5, 5.0 and 50 *μ*M) for 48 h. Western blot analysis to detect activated caspases illustrates that Titanocene Y induces the time- and dose-dependent activation of caspase-3 and -7 in A431 cells (Figures 6A and B).

### The general caspase inhibitor, z-VAD-fmk, blocks cell death induced by Titanocene Y and cisplatin in A431 cells

To confirm the involvement of caspases during apoptosis, cells were pretreated with the pan-caspase inhibitor, z-VAD-fmk, prior to Titanocene Y or cisplatin, and apoptosis was determined by flow cytometry following propidium iodide staining. Titanocene Y and cisplatin alone induced 18 and 23% apoptosis, respectively. However, pretreatment with z-VAD-fmk inhibits apoptosis induced by Titanocene Y and cisplatin in A431 cells, confirming that apoptosis occurs through a caspase-dependent pathway and is in agreement with previous reports that z-VAD-fmk blocked apoptosis induced by cisplatin in A431 cells (Sawada *et al*, 2003).

## DISCUSSION

Despite the widespread use of cisplatin as an antitumour agent, its remarkable antitumour effects coincide with two major drawbacks that have markedly limited cisplatin-based chemotherapy. These include the marked toxic effects, such as neurotoxicity, nephrotoxicity, severe emesis and the development of resistance to platinum-based drugs (Siddik, 2003). Mechanisms of drug resistance limit DNA damage through reduced drug uptake, increased drug inactivation and increased DNA adduct repair. In addition, tumour cells develop mechanisms to overcome death signals, including the loss of p53 function, overexpression of antiapoptotic Bcl-2 and interference with caspase activation (Gonzalez *et al*, 2001; Siddik, 2003).

The development of new organometallic complexes with lower toxicity and improved activity is important for improved patient treatment options (Reedijk, 2003). In addition, alternative DNA damage-signalling pathways need to be evoked to circumvent the development of drug resistance. This observation has led to the synthesis of novel [(1,2-diaryl-1,2-dicyclopentadienyl)ethanediyl] titanium dichlorides (Tacke *et al*, 2004), which combine the reactivity of the titanium dichloride moiety with the ability of hydrogen bonding towards DNA of the amine ligand of cisplatin, if the aryl group is substituted accordingly. We have shown that one member from this series, Titanocene X is significantly more effective than titanium dichloride and exhibits potent cytotoxic effect on kidney carcinoma cells and is cytotoxic to cisplatin-resistant human ovarian carcinoma (Kelter *et al*, 2005). The antitumour potential of novel titanocenes was further demonstrated in freshly explanted human cervical carcinoma cells and kidney cells (Oberschmidt *et al*, 2005), xenografted Caki-1-bearing mice (Fichtner *et al*, 2006), xenografted Ehrlich's ascites tumours (Valadares *et al*, 2006) and MCF-7 xenografted mouse model of breast cancer (Beckhove *et al*, 2007). The dose-dependent effects observed in these *in vivo* models of human disease demonstrate the lack of general toxicity exerted by highly substituted titanocenes in tumour cells and suggest a tumour-specific effect. Furthermore, previous studies indicate that titanocenes have potent, broad spectrum antiproliferative activity against a range of human tumour cells, including multidrug-resistant cells and cells that overexpress the antiapoptotic Bcl-2, therefore suggesting the potential of this novel range of compound for treating drug-refractory cancers (Kelter *et al*, 2005; O'Connor *et al*, 2006). In the current study, we have demonstrated that titanocenes cause dose- and time-dependent antiproliferative effects in HeLa and A431 cells. The most significant results were observed with Titanocene Y, which was more effective than cisplatin at reducing proliferation of A431 cells. On the basis of this observation, the effect of Titanocene Y and cisplatin was investigated in an A431 xenograft mouse model. Results clearly illustrate that Titanocene Y is as effective as cisplatin at reducing tumour cell growth *in vivo* at their respective maximum tolerable dose, when compared to solvent-treated animals. In addition, there was no significance difference in loss of body weight in animal groups treated with the two compounds.

Cisplatin induces a combination of apoptosis and necrosis in human tumour cells (Gonzalez *et al*, 2001; Reedijk, 2003). Necrosis may occur directly as a result of cisplatin treatment depending on the level of cellular damage induced or as a consequence of an unfinished apoptotic programme (Gonzalez *et al*, 2001). Alternatively, the induction of necrosis may reflect the non-specific toxicity often associated with cisplatin treatment. A precise description of the mechanism of action of titanocenes is important to determine their suitability in the treatment of various tumour cells in addition to their suitability in combination treatment regimens. We have confirmed that Titanocene Y-induced cell death occurs via apoptosis as shown by Annexin V-positive staining.

The precise mechanism of action of agents such as cisplatin and its analogues is not fully understood; however, it is closely related to their DNA-binding effects. Cellular effects include inhibition of DNA synthesis and RNA transcription, which leads to the activation of cell cycle checkpoints and transient S-phase arrest followed by a durable G_2_/M arrest (Shi *et al*, 1994; Shapiro and Harper, 1999; Gonzalez *et al*, 2001; Reedijk, 2003; Siddik, 2003). In this study, we investigated the cell cycle effects of novel Titanocenes X and Y. Consistent with previous reports, cisplatin induced S-phase arrest of A431 cells, which preceded apoptosis as shown by the appearance of a sub-G_1_ peak. Cp_2_TiCl_2_ failed to induce any significant cell cycle alterations or cell death in A431 or HeLa cells at the concentration used, whereas Titanocene X caused a small increase of cells in G_2_/M, with a concomitant induction of apoptosis. However, the most potent compound, Titanocene Y induced early G_2_/M arrest of A431 cells, which was followed by a significant increase in apoptotic cells. In addition to A431 cells, Titanocene Y and cisplatin induced apoptosis in HeLa cervical carcinoma cells. These results clearly illustrate the more potent cytotoxic activity of Titanocene Y over Titanocene X. Evaluation of the cell cycle effects observed suggest that the DNA damage initiated by cisplatin and titanocenes results in the activation of diverse cell cycle response. An understanding of the mode of action of titanocenes is important in refining the therapeutic approaches that further enhance the antitumour activity of these compounds. This understanding is also critical for elucidating the mechanisms underlying the development of drug resistance.

Although cisplatin-induced apoptosis occurs through caspase-dependent and caspase-independent mechanisms (Gonzalez *et al*, 2001), caspase-dependent apoptosis has been reported for the A431 cell line (Mese *et al*, 2000; Sawada *et al*, 2003). We examined the involvement of caspases during Titanocene Y-induced apoptosis in A431 cells. Caspase-3 and -7 are important downstream executioners of intrinsic and extrinsic apoptotic pathways, whereas caspase-8 is an upstream caspase that plays a key role during death receptor-mediated apoptosis (Kasibhatla and Tseng, 2003; Vermeulen *et al*, 2005). We found that caspase-3 and -7 are activated in a dose- and time-dependent manner during titanocene-induced apoptosis in A431 cells; however, we could not detect activation of caspase-8. The z-VAD-fmk pan-caspase inhibitor prevented apoptosis induced by cisplatin and Titanocene Y, confirming the importance of caspases in the cell death process. These results are consistent with the activation of an intrinsic non-receptor-mediated apoptotic pathway in A431 cells. However, these findings are in contrast to a previous report of caspase-independent cell death induced by Titanocene Y in prostate cancer cells (O'Connor *et al*, 2006), suggesting that titanocenes are capable of inducing a number of cell death pathways. The precise death pathway initiated appears to be cell type-specific and may depend on the extent of the initial DNA damage induced in each cell type. The specificity of titanocenes for apoptotic cell death may represent an important advantage of these compounds over cisplatin in cancer therapy.

Overall, we have described a new family of compounds that show promising *in vitro* and *in vivo* antitumour activity and may provide improved treatment options for the management of metastatic and recurrent cancer.

## Figures and Tables

**Figure 1:**
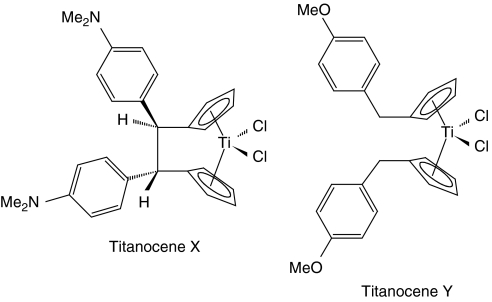
Molecular structures of Titanocenes X and Y.
